# A Comprehensive Polyphenolic Characterization of Five Montmorency Tart Cherry (*Prunus cerasus* L.) Product Formulations

**DOI:** 10.3390/foods14071154

**Published:** 2025-03-26

**Authors:** Muhammad Jawad, Stephen T. Talcott, Angela R. Hillman, Robert G. Brannan

**Affiliations:** 1 Department of Translational Biomedical Sciences, Ohio University, Athens, OH 45701, USA; mj841722@ohio.edu; 2 Department of Food Science & Technology, Texas A&M University, College Station, TX 77845, USA; stephen.talcott@ag.tamu.edu; 3 Department of Exercise Physiology, Ohio University, Athens, OH 45701, USA; hillman@ohio.edu; 4 Department of Food and Nutrition Sciences, Ohio University, Athens, OH 45701, USA

**Keywords:** Montmorency tart cherry (MTC), sour cherry, polyphenols, anthocyanins, flavonols, flavanols, hydroxycinnamic acids, hydroxybenzoic acids

## Abstract

The Montmorency tart cherry (*Prunus cerasus* L., MTC) polyphenols may contribute to reduced inflammation and oxidative stress biomarkers in the body. However, a comprehensive polyphenolic profile of MTC products is lacking. This study provides a comparative analysis of the polyphenolic distribution of individual anthocyanins, flavonols, flavanols, hydroxycinnamic acids, and hydroxybenzoic acids in five MTC products (frozen raw fruit, freeze-dried powder, sweet dried fruit, unsweetened dried fruit, juice concentrate). Twenty-three polyphenols were detected, and 21 were positively identified. Results from three replicates indicate that frozen raw MTC has the most total polyphenolics. Juice concentrate, unsweetened dried MTC, freeze-dried MTC powder, and sweet dried MTC contained 26%, 40%, 60%, and 77% fewer total polyphenolics than frozen raw MTC. Hydroxycinnamic acids, flavonols, and anthocyanins predominated, accounting for 87–99% of total polyphenols in MTC products. Chlorogenic acid, rutin, cyanidin-3-sophoroside, feruloquinic acid, ferulic acid, and coumaric acid isomers were noteworthy polyphenolics. Hydroxycinnamic acids predominated in sweet dried (82%), unsweetened dried (74%), juice concentrate (66%), and frozen-raw (54%) MTC. Flavonols predominated in freeze-dried MTC powder (52%). Anthocyanins, particularly cyanidin glycosides, were important polyphenolics in frozen-raw cherries (18%) but less so in other MTC products. These findings highlight the variability in polyphenols in MTC products and emphasize the importance of selecting appropriate MTC products for specific health benefits.

## 1. Introduction

Polyphenols are naturally occurring bioactive compounds found in plants and plant-based foods, recognized for their potential health benefits, particularly their antioxidative and anti-inflammatory properties. They comprise multiple phenolic structures with hydroxyl groups (-OH) attached to their termini. Polyphenols are crucial in plant defense mechanisms, growth, and development [1,2]. Scientific interest in polyphenols has significantly increased over the past two decades [3].

Studies on the health-promoting effects of polyphenols have established their role in neutralizing free radicals and reducing pro-inflammatory mediators in the body [4]. Among polyphenol-rich fruits, Montmorency tart cherries (*Prunus cerasus* L., MTC) are notable for their rich total polyphenolic content [5], compounds linked to the reduction in oxidative stress and inflammation in the body [6,7,8,9,10]. Research data suggest that regular consumption of MTC may aid in mitigating symptoms of chronic diseases, such as osteoarthritis [11] and cardiovascular diseases [12,13,14,15], by targeting pathways that regulate inflammatory and oxidative responses [16,17].

In MTC, anthocyanins and phenolic acids [18,19] are the predominant polyphenols, and these compounds and research report their benefits in reducing biomarkers of inflammation and oxidative stress, as well as supporting muscle recovery [20,21,22]. Several studies have examined the effects of individual MTC products on health outcomes; for instance, reductions in exercise-induced muscle soreness and inflammation following consumption of MTC juice [23] and enhanced recovery and reduced muscle damage markers among athletes using MTC freeze-dried powder [24] have been reported.

Despite these benefits, the polyphenolic profiles across MTC products are often incomplete, focusing on specific polyphenolic compounds or subclasses, particularly anthocyanins [18,19,25]. Further, processing methods such as heating [26,27,28], drying [29,30,31,32], and freeze-drying [33,34,35] can alter the polyphenolic content and, consequently, the efficacy of these products. Given these factors, a comprehensive comparison of polyphenolic profiles across various MTC products is warranted because there is a paucity of direct comparisons of polyphenolic content in commercially available products. Therefore, the objective of this study was to use LC–MS-MS to determine the polyphenolic profiles of five MTC products (frozen raw fruit, juice concentrate, freeze-dried powder, dried sweetened, and dried unsweetened) available in the market, facilitating direct comparison of polyphenolic compounds to inform the selection of optimal MTC products for health-related applications.

## 2. Materials and Methods

### 2.1. Materials

Five different commercially available MTC products (frozen raw fruit, freeze-dried powder, sweet dried, unsweetened dried, and juice concentrate) were procured from King Orchards, Central Lake, MI, USA. All products were manufactured using proprietary processes from MTC harvested in 2022, except for the concentrate from cherries harvested in 2023. The sweet dried cherries contained 5% added sugar based on dry weight. Except for the minimal use of processing aides (<1%) in the dried products, no other ingredients were added to the products. Solvents were purchased from Thermo Fisher. External standards (catechin, chlorogenic acid, cyanidin-3-glucoside, cyanidin-3-rutinoside, (-)-epicatechin, *p*-coumaric acid, caffeic acid, ferulic acid, gallic acid, naringenin, procyanidin B2, protocatechuic acid, quercetin, rutin, kaempferol, melatonin, cyanidin-3-sophoroside, peonidin, and *o*-coumaric acid) were obtained from Phytolab, Vestenbergsgreuth, Germany or Sigma Aldrich, St. Louis, MO, USA.

### 2.2. Sample Extraction

To prepare the extracts for LC–MS-MS analysis, MTC frozen raw fruit, freeze-dried powder, sweet dried fruit, and unsweetened dried fruit (1 g) were each combined with methanol acidified with 0.1% formic acid (10 mL). Each mixture was thoroughly homogenized for 10 min, followed by 30 min of sonication to ensure optimal extraction. The fruit extracts were centrifuged at 12,000× *g* for 30 min to obtain supernatants that were filtered using 0.22 µm PTFE filters and transferred into HPLC vials for analysis. For the tart cherry juice preparation, 1 part of MTC juice concentrate was first diluted with seven parts distilled water, and the same preparation procedure followed. All prepared MTC fruit extracts were stored at −10 °C until further analysis by LC–MS–MS. Three replicates were performed.

### 2.3. Triple Quadrupole Liquid Chromatography–Mass Spectrophotometry (LC–MS–MS)

Analysis was conducted on a Thermo Scientific Vanquish^TM^ UHPLC equipped with a Thermo Scientific TSQ Altis triple quadrupole mass spectrometer with a HESI source (Waltham, MA, USA). The mobile phases included 0.1% formic acid in mass spectroscopic grade water (Solvent A) as the aqueous phase and 0.1% formic acid in mass spectroscopic grade methanol (Solvent B) as the organic phase. The analysis was carried out over a 10-minute run using a Waters SunFireTM C18 column (150 × 3 mm, 5 μm, Milford, MA, USA) set at 25 °C with a 1 µL injection volume and a flow rate of 0.4 mL/min. The gradient began at 95% Solvent A:5% solvent B and was run from 0 to 8 min in a linear gradient to 5% Solvent A:95% solvent B and held at 95% Solvent B for an additional 2 min prior to equilibration to initial conditions.

MS data were acquired in either negative or positive ESI using Chromeleon 7.2.10 ES software based on optimal ionization conditions of each compound. The parameters consisted of a 3913 V spray voltage, nitrogen gas as the sheath gas at 24 arbitrary units, argon as the auxiliary gas at 2 arbitrary units, ion transfer tube temperature at 325 °C, and the vaporizer temperature at 350 °C. Compound identification was accomplished by comparing retention times, ionization fragmentation patterns, and relative ion abundances with known analytical standards and through online database (www.hmdb.ca) searches. Quantification was performed using calibration curves obtained from analytical standards or in equivalence of a structurally similar standard and when an authentic standard was unavailable.

## 3. Results and Discussion

The polyphenolic compounds and their LC–MS–MS characteristics across the five MTC products are shown in Table 1. Twenty-one of the 23 polyphenolic compounds were identified based on retention times, molecular weights, deprotonated ion masses, and fragment ions compared to authentic standards. Two others, taxifolin and feruloquinic acid, were not able to be positively identified but were semi-quantified based on their structurally similar compounds, quercetin and ferulic acid, respectively. Taxifolin was identified with a molecular weight of *m/z* 303.03 in negative ion mode with fragment ions at *m/z* 124.97, 177.04, and 285.04. Similarly, feruloquinic acid was detected with a molecular weight of *m/z* 367 (*m/z*) in negative ion mode and fragment ions at *m/z* 134.04, 190.95, and 193.04.

### 3.1. Polyphenolics in MTC Products

The predominant polyphenolic classes in the five MTC products are shown in Table 2. Total phenolics summed across all the individual polyphenols indicate that frozen raw MTC contained the most total polyphenolics of the five MTC products. MTC juice concentrate, unsweetened dried MTC, freeze-dried MTC powder, and sweet dried MTC contained 26%, 40%, 60%, and 77% fewer total polyphenolics than the frozen raw MTC. This result suggests that processing has an impact on polyphenolic levels in MTC products, and there is evidence that polyphenols degrade during processing, especially drying [18,36,37,38]. However, this study is limited to a survey of five commercially available MTC products, so unknown factors such as genetic variability, agricultural practices, post-harvest handling and storage, and processing conditions make systematic conclusions unreliable.

All five MTC products were similar in that hydroxycinnamic acids, flavonols, and anthocyanins predominated, accounting for 87–99% of the total polyphenols in each product. Hydroxycinnamic acids were predominant in sweet dried MTC, unsweetened dried MTC, MTC juice concentrate, and frozen raw MTC, accounting for 83%, 74%, 66%, and 54% of total polyphenolics, respectively. Of the hydroxycinnamic acids, chlorogenic acid predominated, but feruloquinic acid, ferulic acid, and coumaric acid isomers were notable in certain MTC products (Table 3). Although freeze-dried cherry powder contained 29% hydroxycinnamic acids, flavonols predominated and accounted for 52% of total polyphenolics. Flavonols accounted for 12–19% of the other four MTC products, and rutin (quercetin-3-O-rutinoside) was the predominant flavonol in all MTC products. Anthocyanins, particularly cyanidin glycosides, were important polyphenolic compounds in frozen raw cherries, where they accounted for 18% of total polyphenolics, but less so in the other four MTC products, where they accounted for 4–6% of total polyphenolics.

#### 3.1.1. Hydroxycinnamic Acids

Seven hydroxycinnamic acids and their glycosides were detected in the MTC products (Table 1), and although the identity of feruloquinic acid could not be confirmed because it was not compared to an authentic standard, spectroscopic clues lent confidence to its inclusion in this discussion. Chlorogenic acid and feruloquinic acid in an approximately 3:1 ratio were the predominant hydroxycinnamic compounds in four of the MTC products, whereas chlorogenic acid, feruloquinic acid, and ferulic acid accounted for the hydroxycinnamic acids in a nearly equal 1:1:1 ratio in freeze-dried MTC powder. The two coumaric acid isomers (*p*- and *o*-) were found collectively at 8–17% of hydroxycinnamic acids in the MTC products. Caffeic acid (4%) and its glycoside did not account for more than 2% of the hydroxycinnamic acids in any MTC products except MTC juice concentrate (4% caffeic acid).

Compared to the frozen raw MTC, there were fewer hydroxycinnamic acids in the other four MTC products, especially in the sweet dried cherries (65% fewer) and freeze-dried powder (79% fewer). This result likely was due to the presence of less chlorogenic acid, feruloquinic acid, and ferulic acid in the MTC products compared to the frozen raw MTC.

Hydroxycinnamic acids, including caffeic acid, ferulic acid, chlorogenic acid, and *p*-coumaric acid, play an important role in neutralizing free radicals [39,40]. This action helps reduce oxidative stress and protects cells from damage associated with chronic diseases such as cardiovascular disease by preventing LDL oxidation [41], a key factor in atherosclerosis development, and enhancing blood vessel function [42]. Additionally, hydroxycinnamic acids exhibit anti-inflammatory properties by modulating inflammatory pathways, which can be beneficial for conditions like arthritis [43]. They also possess antimicrobial properties and may enhance gut health by promoting beneficial bacteria [44].

#### 3.1.2. Flavonols

Four flavonols, also known as 3-hydroxyflavones, were identified across the five MTC products (Table 1). Rutin (quercetin-3-O-rutinoside) accounted for 95–98% of the total flavonols (Table 3) and was the predominant flavonoid in each of the MTC products regardless of flavonoid subclass. Compared to the frozen raw MTC, less rutin was identified in MTC juice concentrate (37% less), unsweetened dried MTC (40% less), and especially sweetened dried MTC (86% less). However, freeze-dried MTC was identified at the same level as frozen raw MTC. This suggests that lyophilization either exerts a protective mechanism on rutin or enhances its extractability [45,46].

Three other flavonols were detected in the MTC products. Small amounts of either quercetin or its glucoside were identified in each MTC product; however, the amounts that were identified were very near to the lower detection limit of the assay and together accounted for 2.0%, 0.5%, 0.5%, 0.4%, and 0.2% of all flavonoids in freeze-dried, juice concentrate, unsweetened dried, frozen raw, and sweet dried MTC, respectively. While the presence of free quercetin in tart cherry has been reported in the literature [18,47], it is also possible that its detection may result from enzymatic or other degradation of quercetin 3-glucoside. Kaempferol-3-rutinoside was detected in very small amounts in all MTC products and accounted for 0.1–0.5% of all flavonoids in the MTC products.

Flavonols are well-recognized for their ability to mitigate oxidative stress, which may help decrease the risk of chronic diseases such as cardiovascular disease [48,49] and cancer [50,51]. Rutin is a potential natural senomorphic agent with therapeutic potential for age-related diseases, including cancer [52,53], reducing inflammation [54], increasing antioxidant capacity [55], and improving gut morphology and gut microflora [56]. Rutin and quercetin have also demonstrated preventive effects against DXR-induced hepatotoxicity via inhibiting inflammation, oxidative stress, and apoptosis with attenuating Nrf2 expression in rats [57]. Quercetin is found to be associated with improved cardiovascular health by reducing blood pressure [58,59,60] and inhibiting platelet aggression [61,62]. Similarly, kaempferol has shown potential in reducing oxidative damage [63,64] and inflammation [64,65], making it a viable agent for antioxidative and anti-inflammatory therapies.

MTC products emphasize how processing methods can affect flavonol compounds. Some products contain substantial levels of flavonol compounds, while others, like frozen raw fruit and sweet dried cherries, may have concentrations below detectable thresholds. This may suggest that concentrating or drying MTC in the absence of sugar enhances the protective effect on flavonols. The potential of sugars to promote the degradation of polyphenols, including anthocyanins and potentially flavonols, through Maillard reactions or by altering their thermal stability has been reported [66].

#### 3.1.3. Anthocyanins

Of the five anthocyanins identified in MTC (Table 1), only the cyanidin glycosides were detected in all MTC products, but their distribution was dependent on the product. Cyanidin-3-sophoroside was the predominant anthocyanin in frozen raw MTC (65%) and sweet dried MTC (43%). Cyanidin-3-rutinoside (36%) and cyanidin-3-glucoside (21%) accounted for the remaining 57% of anthocyanins in sweet dried MTC, whereas the remaining anthocyanins in raw frozen MTC were distributed evenly between the cyanidin glycosides (17%) and peonidin-3-rutinoside (17%). Cyanidin-3-rutinoside predominated in unsweetened dried MTC, juice concentrate, and freeze-dried MTC powder (59%, 52%, and 42%, respectively), and these products also contained considerable cyanidin-3-glucoside (34%, 31%, 25%). The presence of peonidin and its rutinoside was variable in these three MTC products. Unsweetened dried MTC contained only cyanidin glycosides, MTC juice concentrate contained peonidin-3-rutinoside (10%), and freeze-dried MTC powder contained 10% peonidin-3-rutinoside (15%) and was the only anthocyanidin that contained peonidin aglycone (4%).

Anthocyanins play an important role in reducing oxidative stress, which may help lower the risk of cardiovascular diseases by improving blood vessel flexibility [67,68] and reducing hypertension [13]. Additionally, anthocyanins possess anti-inflammatory properties that are particularly beneficial for managing osteoarthritis [69,70,71], as they can help reduce inflammation and alleviate pain. Emerging research also indicates that anthocyanins support brain health by protecting neurons from oxidative damage, potentially enhancing cognitive function, and decreasing the risk of neurodegenerative diseases [72].

#### 3.1.4. Flavanols (Flavan-3-ols)

Four flavan-3-ols were identified in the five MTC products (Table 1), and although the identity of taxifolin could not be confirmed because it was not compared to an authentic standard, spectroscopic clues lent confidence to its inclusion in this discussion. Taxifolin, which has the basic structure of a flavan-3-ol with a ketone at the 4-position of its C-ring, is sometimes considered to be in the flavonoid/flavan-3-ol subclass of flavanonols; however, herein, it is considered together with the flavan-3-ols. Overall, flavan-3-ols account for only a fraction of total polyphenolics in the frozen raw MTC (7%), MTC juice concentrate (11%), and freeze-dried MTC powder (2%). The amount and distribution of (-)-epicatechin, catechin, and procyanidin B2 were nearly identical in frozen raw and juice concentrate MTCs, and both MTC products contained minimal (1–2%) taxifolin. The distribution of these flavan-3-ols was similar in freeze-dried MTC powder; however, it contained 15-fold less (-)-epicatechin, 18-fold less catechin, and 29-fold less procyanidin B2. There were almost no flavan-3-ols (0.1%) detected in both the unsweetened and sweetened dried MTC, a result that has been previously reported [73].

The absence of larger procyanidin polymers in the MTC products is noteworthy and aligns with the previous study on tart cherries, which reported that tart cherries predominantly contain short-chain procyanidins, with an average polymerization degree of four monomer units and lack the high concentrations of long-chain procyanidins observed in other cherry species [74]. Although targeted LC–MS-MS did not detect significant amounts of larger flavan-3-ol polymers in MTC products, it is possible that these compounds exist in higher concentrations but are bound to proteins or anthocyanins. This binding may form high-molecular-weight complexes that are difficult to extract and analyze. Previous studies have shown that flavan-3-ols can interact with proteins and anthocyanins, leading to the formation of new polymeric species that require advanced fractionation techniques for detection [75,76,77].

After ingestion, flavan-3-ols undergo metabolic transformations, with secondary metabolites subsequently detected in blood plasma, where they have the potential to exert systemic health effects. Research has demonstrated that catechin and (-)-epicatechin support cardiovascular health by enhancing endothelial function and reducing blood pressure, attributable to their vasodilatory and anti-inflammatory properties [78,79]. Procyanidin B2, although present in lower concentrations, exhibits potent antioxidant effects, particularly in mitigating oxidative stress, and is associated with benefits for skin [80,81] and vascular health through its role in neutralizing free radicals [82,83]. Collectively, these polyphenols contribute to decreased oxidative stress and inflammatory markers, supporting their role in the management of inflammatory conditions when consumed regularly.

#### 3.1.5. Flavanones

Naringenin is the lone flavanone detected in the MTC products (Table 1) and only accounted for only 3% of polyphenolics in freeze-dried MTC powder and less than 1% in the other MTC products. Research has shown that naringenin can reduce oxidative stress by neutralizing free radicals, which helps protect cells from damage associated with aging and various chronic diseases [84,85]. Additionally, studies also suggest that naringenin may improve cardiovascular health by improving lipid levels [86,87] and alleviating arthritis symptoms [88,89]. Moreover, naringenin plays a significant role in inhibiting the growth of breast [90,91] and prostate cancer cells [92] through processes such as cell apoptosis and regulation of the cell cycle.

#### 3.1.6. Hydroxybenzoic Acids

Gallic acid and protocatechuic acid were the hydroxybenzoic acids detected and quantified in the MTC products (Table 1); however, only freeze-dried MTC powder contained hydroxybenzoic acids at a substantial level (8%). The amount of hydroxybenzoic acids in the other MTC formulations was 1% or less. In freeze-dried MTC powder, the gallic acid to protocatechuic acid ratio was 10:1. It is not clear from these data if the lack of hydroxybenzoic acids in the MTC products that were not freeze-dried was due to natural variation or some other cause. Hydroxybenzoic acids are known as antioxidants that can neutralize free radicals, reduce oxidative stress, and prevent cellular damage. Gallic acid has demonstrated anti-inflammatory effects [93], which contribute to its ability to reduce inflammation and the risk of cardiovascular diseases [94]. Protocatechuic acid is associated with neuroprotective effects, helping to prevent neurodegenerative diseases by shielding neuronal cells from oxidative stress [95].

### 3.2. Summary

The polyphenolic profiling presented in this study offers a more comprehensive analysis of the array of polyphenols in MTC products compared to previous research, which is often limited to specific polyphenolic compounds or classes [18,19,25]. For example, Kim et al. (2005) focused on phenolic content and anthocyanin stability in sweet and sour cherry cultivars but limited the analysis to a few major compounds, particularly cyanidin-3-glucoside [25]. Kirakosyan et al. (2009) analyzed similar products to those of the current study but focused on anthocyanins and a few other selected flavonoids without in-depth polyphenol quantification across the products [18]. Ou et al. (2012) explored polyphenols in several forms and emphasized anthocyanins, procyanidins, and antioxidant capacity without covering the full array of individual polyphenols [19].

In contrast, this study evaluated five distinct MTC products (frozen raw fruit, freeze-dried powder, sweet dried fruit, unsweetened dried fruit, and juice concentrate) and provided an extensive comparative analysis of polyphenolic distribution that includes anthocyanins, flavonols, flavanols, hydroxycinnamic acids, and hydroxybenzoic acids. The analysis confirms the presence of commonly studied compounds, such as the cyanidin derivatives, and quantifies lesser-examined components like caffeic acid glycoside and peonidin-3-rutinoside.

Specifically, twenty-three polyphenols were detected, and 21 were positively identified, and the results show that frozen raw MTC has the most total polyphenolics. Juice concentrate, unsweetened dried MTC, freeze-dried MTC powder, and sweet dried MTC contained 26%, 40%, 60%, and 77% fewer total polyphenolics than the frozen raw MTC. Hydroxycinnamic acids, flavonols, and anthocyanins predominated, accounting for 87–99% of total polyphenols in MTC products. Chlorogenic acid, rutin, cyanidin-3-sophoroside, feruloquinic acid, ferulic acid, and coumaric acid isomers were the noteworthy individual polyphenolics. Hydroxycinnamic acids predominated in sweet dried MTC (82%), unsweetened dried MTC (74%), juice concentrate (66%), and frozen-raw (54%) MTC, and flavonols predominated in freeze-dried MTC powder (52%). Anthocyanins, particularly cyanidin glycosides, were important polyphenolics in frozen raw cherries (18%) but less so in other MTC products.

## 4. Conclusions

This comprehensive polyphenolic profiling of MTC products offers insights that impact the practical and theoretical understanding of these commercial items. Practically, fewer total polyphenolics were observed in processed forms compared to frozen raw MTC, which underscores the importance of processing methods in preserving these compounds. These data allow consumers and manufacturers to make informed decisions regarding product selection and processing techniques, potentially favoring less processed options to maximize polyphenol intake. Theoretically, this work differs from prior work that often focused on limited subsets of polyphenols and revealed that frozen raw MTC exhibited the highest total polyphenolic content. The quantification of less frequently examined compounds, such as caffeic acid glycoside and peonidin-3-rutinoside, alongside the confirmation of more commonly studied cyanidin derivatives, offers the possibility of a more detailed understanding of the polyphenolic profile in MTC. This foundation can be used as justification for clinical trials with MTC polyphenol-rich products to establish their health benefits and bioavailability, which would help to understand how well MTC polyphenols are metabolized and absorbed in the body to demonstrate their beneficial effects.

## Figures and Tables

**Table 1:** Characterization of polyphenolic compounds in Montmorency tart cherry using triple quadrupole LC–MS-MS, sorted by molecular weight (MW).

Phenolic Compounds	Phenolic Class	RT (min)	MW (*m*/*z*)	[M-H]^−^ (*m*/*z*)	MS/MS (*m*/*z*)	Confirmation
Protocatechuic acid	Hydroxybenzoic Acid	2.24	154.12	153.05	81.05, 91.04, 109.04	Identified
*p*-Coumaric acid	Hydroxycinnamic Acid	3.00, 3.40	164.04	162.95	93.07, 117.00, 119.04	Identified
o-Coumaric acid	Hydroxycinnamic Acid	3.03, 4.40	164.16	162.93	93.04, 117.04, 119.00	Identified
Gallic acid	Hydroxybenzoic Acid	6.56	170.12	168.83	79.11, 97.04, 125.22	Identified
Caffeic acid	Hydroxycinnamic Acid	3.61	180.16	178.99	89.07, 107.07, 135.07	Identified
Ferulic Acid	Hydroxycinnamic Acid	4.62	194.18	192.97	134.04, 149.11, 178.04	Identified
Naringenin	Flavonol	4.95, 6.05	272.25	270.99	107.04, 119.07, 151.04	Identified
Catechin	Flavanol (Flavan-3-ol)	3.69	290.26	289.04	203.07, 204.97, 245.04	Identified
(-)-Epicatechin	Flavanol (Flavan-3-ol)	3.69	290.26	289.39	187.54, 203.69, 245.29	Identified
Peonidin	Anthocyanin	5.74	301.27	299.87	259.07, 202.12, 287.12	Identified
Quercetin	Flavonol	5.98	302.23	301.09	151.04, 179.04, 121.04	Identified
Taxifolin * (dihydroquercetin)	Flavanol (Flavan-3-ol) *	4.4	304.25	303.03	124.97, 177.04, 285.04	Unconfirmed
Caffeic acid glycoside	Hydroxycinnamic Acid	3.36	342.30	341.00	131.01, 179.04, 220.92	Identified
Chlorogenic acid	Hydroxycinnamic Acid	2.52, 3.35	354.31	353.31	191.05, 351.07, 354.09	Identified
Feruloquinic acid	Hydroxycinnamic Acid	3.27	368.34	367.00	134.04, 190.95, 193.04	Unconfirmed
Quercetin-3-glucoside	Flavonol	4.96	463.40	462.86	255.13, 271.08, 299.77	Identified
Cyanidin-3-glucoside	Anthocyanin	4.65, 5.30	484.83	447.18	255.08, 284.13, 285.13	Identified
Procyanidin B2	Flavanol (Flavan-3-ol)	3.00, 3.34	578.52	577.53	289.07, 425.08, 455.07	Identified
Kaempferol-3-rutinoside	Flavonol	5.31	594.52	593.00	285.00, 447.00, 535.00	Identified
Cyanidin-3-rutinoside	Anthocyanin	3.77, 5.30	595.52	594.20	163.06, 307.10, 325.11	Identified
Peonidin-3-rutinoside	Anthocyanin	3.98	609.60	608.00	145.05, 163.06, 307.10	Identified
Rutin (quercetin-3-O-rutinoside)	Flavonol	4.91	610.51	609.00	254.97, 271.04, 300.04	Identified
Cyanidin-3-sophoroside	Anthocyanin	0.16	611.50	610.50	535.21, 488.21, 575.20	Identified
Protocatechuic acid	Hydroxybenzoic Acid	2.24	154.12	153.05	81.05, 91.04, 109.04	Identified

* Taxifolin also can be classified as a flavanonol, sometimes considered a flavonoid subclass distinct from flavan-3-ols.

**Table 2:** Total polyphenolic content of Montmorency tart cherry polyphenol classes in five tart cherry products reported as ppm (dry weight basis) ± standard deviation (*n* = 3) and percentage of each class in each product.

Polyphenol Class	Frozen Raw Cherries	Cherry Juice Concentrate	Unsweetened Dried Cherries	Freeze Dried Cherry Powder	Sweet Dried Cherries
Hydroxycinnamic Acids	44,827 ± 197 (54%)	40,476 ± 16 (66%)	36,296 ± 15 (74%)	9585 ± 17 (29%)	15,519 ± 60 (83%)
Flavonols	16,390 ± 15 (20%)	10,504 ± 2 (17%)	9825 ± 4 (20%)	16,912 ± 11 (52%)	2340 ± 1 (13%)
Anthocyanins	15,204 ± 36 (18%)	2852 ± 3 (5%)	2171 ± 8 (4%)	2060 ± 2 (6%)	691 ± 4 (4%)
Flavanols (Flavan-3-ols)	6107 ± 7 (7%)	6863 ± 9 (11%)	57 ± 1 (<1%)	911 ± 4 (2%)	16 ± 1 (<1%)
Flavanones	254 ± 3 (<1%)	262 ± 1 (<1%)	356 ± 1 (<1%)	928 ± 1 (3%)	79 ± 1 (<1%)
Hydroxybenzoic Acids	101 ± 1 (<1%)	480 ± 2 (<1%)	572 ± 1 (1%)	2531 ± 24 (8%)	161 ± 1 (<1%)

**Table 3:** Individual polyphenolic compounds (ppm dry weight ± standard deviation, n = 3) of Montmorency tart cherry in five tart cherry products.

Polyphenol Compounds	Frozen Raw Cherries	Cherry Juice Concentrate	Unsweetened Dried Cherries	Freeze Dried Cherry Powder	Sweet Dried Cherries
Hydroxycinnamic Acids					
Chlorogenic Acid	29,392 ± 28	22,729 ± 7	21,362 ± 7	2776 ± 7	9544 ± 9
Feruloquinic Acid	8087 ± 54	7226 ± 28	5421 ± 25	2906 ± 12	2383 ± 13
Ferulic Acid	3067 ± 15	2582 ± 33	2456 ± 14	2588 ± 29	1445 ± 26
*p*-Coumaric acid	1750 ± 8	3002 ±1	3018 ± 2	431 ± 0	850 ± 1
o-Coumaric acid	1759 ± 1	3237 ± 3	3256 ± 6	715 ± 3	1031 ± 12
Caffeic acid glycoside	453 ± 0	70 ± 0	42 ± 0	48 ± 0	99 ± 0
Caffeic acid	169 ± 1	1631 ± 0	745 ± 0	133 ± 0	168 ± 0
Flavonols					
Rutin (quercetin-3-O-rutinoside)	15,940 ± 11	10,097 ± 1	9522 ± 3	16,080 ± 9	2292 ± 0
Quercetin-3-glucoside	299 ± 2	203 ± 0	166 ± 0	436 ± 0	31 ± 0
Kaempferol-3-rutinoside	149 ± 0	87 ± 0	75 ± 0	174 ± 0	17 ± 0
Quercetin	nd	117 ± 0	63 ± 0	223 ± 0	nd
Anthocyanins					
Cyanidin-3-Sophoroside	9930 ± 18	207 ± 2	154 ± 5	279 ± 4	302 ± 2
Peonidin-3-rutinoside	2630 ± 16	273 ± 0	nd	313 ± 0	nd
Cyanidin-3-Rutinoside	1667 ± 5	1494 ± 1	1273 ± 3	872 ± 1	245 ± 0
Cyanidin-3-Glucoside	981 ± 1	879 ± 0	745 ± 0	514 ± 4	144 ± 0
Peonidin	nd	nd	nd	84 ± 2	nd
Flavanols (Flavan-3-ols)					
(-)-Epicatechin	2614 ± 15	2665 ± 1	nd	361 ± 2	nd
Catechin	2157 ± 13	2740 ± 6	nd	272 ± 1	nd
Procyanidin B2	1291 ± 15	1323 ± 1	22 ± 0	89 ± 0	nd
Taxifolin (dihydroquercetin)	43 ± 0	136 ± 0	36 ± 0	188 ± 0	16 ± 0
Flavanones					
Naringenin	255 ± 2	262 ± 1	356 ± 1	929 ± 0	79 ± 0
Hydroxybenzoic Acids					
Protocatechuic acid	102 ± 1	480 ± 2	573 ± 1	219 ± 0	161 ± 0
Gallic acid	nd	nd	nd	2311 ± 24	nd

nd = not detected.

## Data Availability

The original contributions presented in the study are included in the article, further inquiries can be directed to the corresponding author.
